# Analysis of serum adiponectin and leptin in patients with acute exacerbation of idiopathic pulmonary fibrosis

**DOI:** 10.1038/s41598-019-46990-3

**Published:** 2019-07-19

**Authors:** Noriyuki Enomoto, Yoshiyuki Oyama, Hideki Yasui, Masato Karayama, Hironao Hozumi, Yuzo Suzuki, Masato Kono, Kazuki Furuhashi, Tomoyuki Fujisawa, Naoki Inui, Yutaro Nakamura, Takafumi Suda

**Affiliations:** 1 Second Division, Department of Internal Medicine, Hamamatsu University School of Medicine, Hamamatsu, Japan; 2 Health Administration Center, Hamamatsu University School of Medicine, Hamamatsu, Japan; 3 Department of Clinical Pharmacology and Therapeutics, Hamamatsu University School of Medicine, Hamamatsu, Japan

**Keywords:** Prognosis, Diseases

## Abstract

Weight loss progresses with the progression of idiopathic pulmonary fibrosis (IPF), and acute exacerbation of IPF (AE-IPF) frequently occurs in its advanced stage. Adiponectin and leptin are adipokines produced from adipose tissue, and are related to thinness and obesity, respectively. Additionally, these adipokines are implicated in the regulation of inflammation and fibrosis centering on peroxisome proliferator-activated receptor γ (PPARγ). However, the relationship between adiponectin/leptin and AE-IPF remains poorly known. We conducted this study to evaluate levels of serum adiponectin/leptin, and to elucidate the clinical importance of adiponectin and leptin in patients with AE-IPF. Thirty-two patients (39 episodes) who were diagnosed with AE-IPF at our hospital from 1997 to 2016 were retrospectively studied. Serum adiponectin and leptin concentrations were measured with enzyme-linked immunosorbent assay. Patients with AE-IPF showed higher levels of serum adiponectin and leptin than those at initial diagnosis of IPF (p = 0.007 and p = 0.027, respectively). Serum adiponectin/leptin (A/L) ratio was negatively correlated with body mass index at AE-IPF (r = −0.456, p = 0.003) and PaO_2_ before AE-IPF (r = −0.498, p = 0.034), and positively correlated with C-reactive protein at AE-IPF (r = 0.316, p = 0.049). Patients with higher A/L ratios had worse survival than those with lower A/L ratios (log-rank, p = 0.026). Further, in multivariate analysis, serum A/L ratio was a significant prognostic factor in patients with AE-IPF (HR 2.60, p = 0.042). In conclusion, the higher adiponectin/leptin ratio may be associated with a poor prognosis in patients with AE-IPF.

## Introduction

Idiopathic pulmonary fibrosis (IPF) shows a catastrophic prognosis^1^, and one of the leading causes of death in patients with IPF is acute exacerbation of IPF (AE-IPF)^2^. AE occurs more frequently in patients with IPF than in those with other interstitial pneumonias such as connective tissue disease-associated interstitial pneumonia^3,4^, and causes 30–40% of all deaths^5,6^. In patients with IPF, AE more often occurs in the advanced stage of IPF than in its early stages^7^. Patients with IPF lose further weight as the disease progresses. Therefore, the amount of adipose tissue should decrease in the advanced stage of IPF.

Adiponectin and leptin are adipokines produced from adipose tissue and are related to obesity and emaciation. These adipokines have pleiotropic functions, and affect lymphocytes, macrophages, epithelial cells, endothelial cells, and smooth muscle cells^8,9^. Additionally, the regulation of inflammation centering on the transcription factor peroxisome proliferator-activated receptor γ (PPARγ) has been reported in the biology of these adipokines^8,9^. Adiponectin and leptin have the opposite effect on PPARγ expression: adiponectin increases the level of PPARγ and leptin conversely decreases it^8^. Regarding fibrosis, decreased adiponectin levels were recently reported in patients with systemic sclerosis and interstitial pneumonia^10,11^. Furthermore, leptin levels in bronchoalveolar lavage fluid reportedly increased in patients with acute respiratory distress syndrome (ARDS), and higher leptin levels were associated with higher mortality in nonobese patients^12^. In addition, Cao *et al*. reported that plasma leptin levels increased at AE-IPF, and AE-IPF patients with higher leptin levels at AE showed worse prognosis than those with lower levels^13^. These results lead us to hypothesize that not only leptin but also adiponectin, which have opposite effects on PPARγ expression, may be related to pathogenesis of AE-IPF in the advanced stage of IPF. Further, adiponectin levels in patients with AE-IPF and their mechanism of action have been unknown.

In the current study, we retrospectively studied patients with AE-IPF and evaluated serum concentrations of these adipokines. To the best of our knowledge, this is the first study to show the relationship between these two adipokines, and the relationship between adipokines and clinical parameters/prognosis in patients with AE-IPF.

## Results

### Clinical characteristics; laboratory, physiological, radiological findings; and treatments of patients with AE-IPF

Clinical characteristics of all patients (39 episodes) are shown in Table 1 and Supplementary Table S1. Thirty-two patients presented their first AE episode and 7 with their second episode. Median body mass index (BMI) and body surface area (Du Bois formula) were 21.9 and 1.59, respectively. The percent predicted forced vital capacity (FVC) and diffusion lung capacity for carbon monoxide (DL_CO_) within 12 months before AE-IPF onset was moderately impaired (median, 57.6% and 57.1%, respectively). The most frequent IPF severity grades were GAP III (36%), and Japanese Respiratory Society (JRS) grade IV (36%), which is the most severe grade in each grading system. Twenty-four of 39 patients (62%) underwent treatment for IPF before AE-IPF, including corticosteroids, immunosuppressants, and pirfenidone. Thirteen patients (33%) underwent long-term oxygen therapy before AE-IPF. At the diagnosis of AE-IPF, the median number of peripheral blood white blood cells was 9755/*μ*L and the median PaO_2_/FiO_2_ (P/F) ratio was 176. Steroid pulse therapy was administered to all patients. Treatments for AE-IPF began as soon as possible after admission concomitantly with antibiotics administration. The period from admission to beginning treatments for AE showed a median of one day.

**Table 1:** Clinical characteristics, severity of IPF, and treatments in all patients with AE-IPF.

	n = 39 episodes, median (range)
Age, years	69 (50, 84)
Sex, male/female	37/2
Smoking, never/ex/current	3/32/4
Smoking pack years at first AE	35 (0, 80)
Period from IPF-diagnosis to AE, mo	75 (0, 205)
Observation period, mo	62 (2, 205)*
The number of AEs, 1/2	32/7
BMI	21.9 (16.3, 28.9)
Body surface area, m^2^	1.59 (1.38, 1.91)
Peripheral blood WBC at AE, /μL	9755 (1900, 20000)
Peripheral blood neutrophils at AE, /μL	6954 (1539, 18200)
CRP at AE, mg/dL	7.4 (0.9, 23.7)
Serum LDH at AE, IU/L	347 (183, 693)
Serum KL-6 at AE, U/mL	1535 (481, 6404)
Serum SP-D at AE, ng/mL	338 (23, 1330)
P/F ratio at AE	176 (38, 386)
HRCT extent scores at AE (full score: 25)	21 (13, 25)
Period from admission to the beginning of AE treatment, days	1 (0, 17)
Administration of steroid pulse therapy at AE, +/−	40/0
Administration of Immunosuppressants at AE, +/−	28/11
Data before AE**	
FVC, % pred	57.6 (37.5, 89.3)
DL_CO_, % pred	57.1 (33.5, 85.9)
PaO_2_ at rest, Torr	72.8 (49.0, 91.0)
HRCT extent scores (full score: 25)	12.5 (7, 19)
Distance in 6MWT, mo	370 (160, 507)
Minimum SpO_2_ in 6MWT, %	82 (60, 95)
JRS severity grade of interstitial pneumonia, I/II/III/IV/unknown	9/0/12/14/4
The GAP staging system, I/II/III/unknown	7/13/14/5
Preceding treatments for IPF, +/−	24/15
Preceding oxygen therapy, +/−	13/26

^*^Observation period in 32 patients with IPF.

**Pulmonary function tests, severity scores, HRCT, and serum markers were evaluated within 12 months before AE-IPF.

Abbreviations; AE: acute exacerbation, BMI: body mass index, FVC: forced vital capacity, DL_CO_: diffusion lung capacity for carbon monoxide, PaO_2_: partial pressure of arterial oxygen, HRCT: high-resolution computed tomography, 6MWT: 6-minute walk test, SpO_2_: partial oxygen saturation, LDH: lactate dehydrogenase, KL-6: Krebs von den Lungen-6, SP-D: surfactant protein D, JRS: Japanese respiratory society, GAP: gender, age, and physiology, IPF: idiopathic pulmonary fibrosis, WBC: white blood cell, CRP: c-reactive protein, P/F: PaO_2_/FiO_2_.

### Serum adiponectin and leptin levels at the diagnosis of AE-IPF

**Figure 1:**
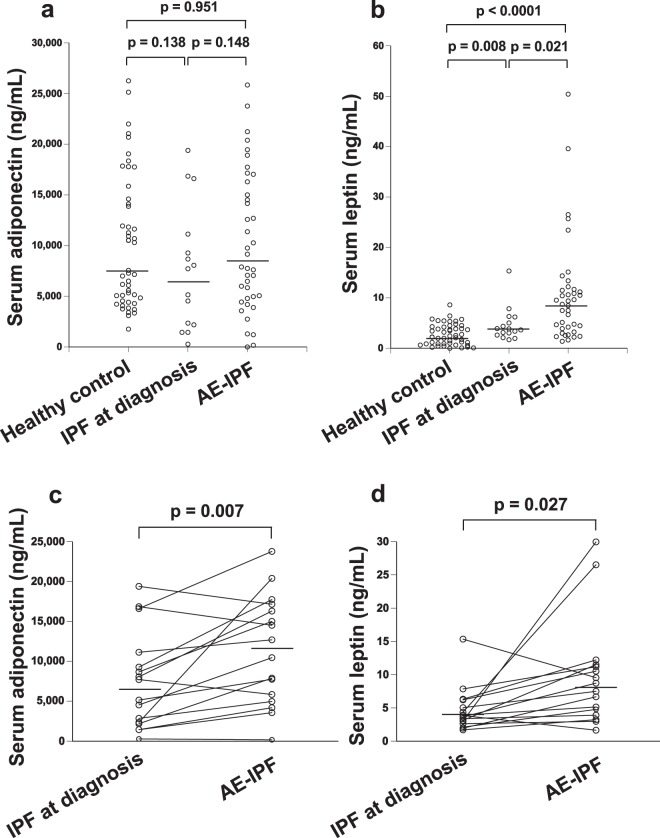
Serum adiponectin and leptin concentrations at the diagnosis of AE-IPF. Serum adiponectin and leptin levels were evaluated in 39 episodes of AE-IPF. Median adiponectin and leptin concentrations at AE-IPF were 7892 ng/mL and 8.72 ng/mL, respectively. Serum adiponectin levels tended to be higher than those at the initial diagnosis of IPF (**a**; p = 0.148). Serum leptin levels at AE-IPF were significantly higher than those at the initial diagnosis of IPF (n = 16) and those of healthy controls (n = 48) (**b**; p = 0.021 and p < 0.0001, respectively). Regarding serial change in identical patients (n = 16), both adiponectin and leptin levels significantly increased at AE-IPF compared with those at initial IPF diagnosis (**c**, p = 0.007; **d**, p = 0.027, respectively).

Serum adiponectin and leptin levels were evaluated in 39 episodes of AE-IPF, for which serum was available at the diagnosis of AE-IPF (Fig. 1). Median adiponectin and leptin concentrations at AE-IPF were 7892 ng/mL and 8.72 ng/mL, respectively. Serum adiponectin level tended to be higher than that at the initial diagnosis of IPF, although the difference was not significant (Fig. 1a, p = 0.148). The serum leptin level at AE-IPF was significantly higher than that at the initial diagnosis of IPF (n = 16) and that in age-matched healthy control (n = 48) (Fig. 1b; p = 0.021 and p < 0.0001, respectively). Regarding serial change in identical patients (n = 16), both adiponectin and leptin levels significantly increased at AE-IPF compared with those at initial IPF diagnosis (Fig. 1c, p = 0.007; Fig. 1d, p = 0.027, respectively).

### Relationships between serum adiponectin/leptin levels and several clinical parameters

**Figure 2:**
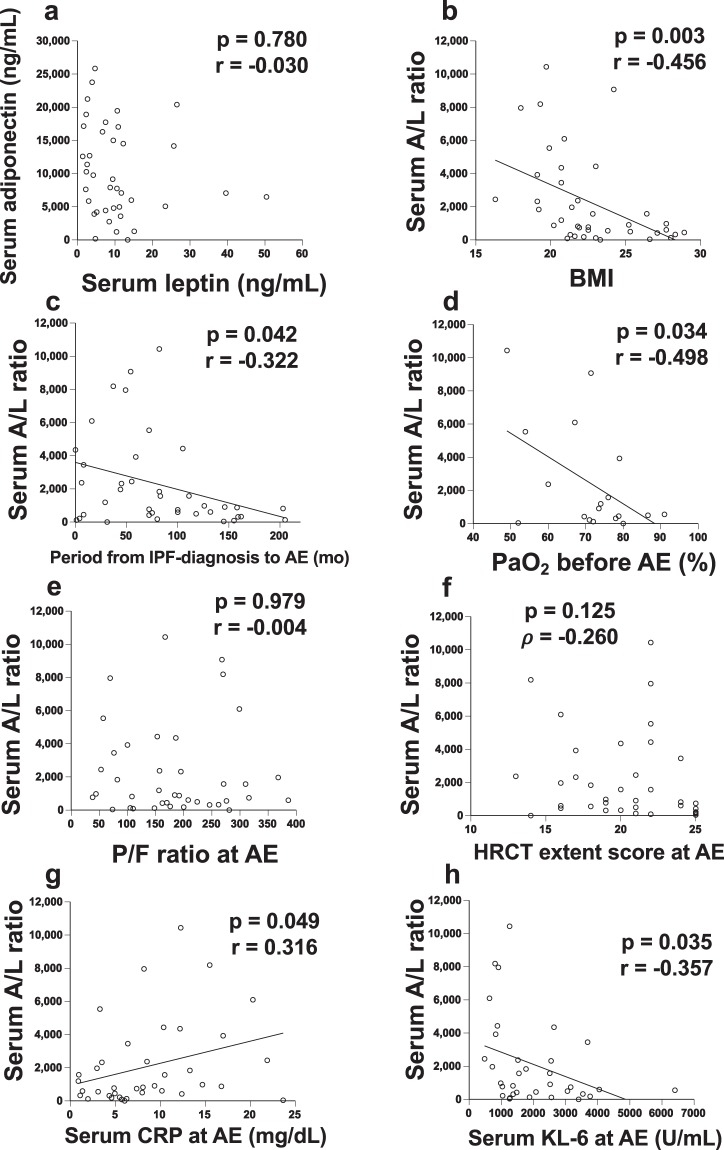
Relationships between serum adiponectin/leptin levels and several clinical parameters. The relationships between serum adiponectin/leptin (A/L) ratio at AE-IPF and clinical parameters are shown. There was no correlation between adiponectin and leptin levels (**a**). A negative correlation was found between A/L ratio and body mass index (BMI) (**b**; p = 0.003, r = −0.456). The A/L ratio was negatively correlated with the period from IPF-diagnosis to AE (**c**; p = 0.042, r = −0.322) and PaO_2_ before AE (**d**; p = 0.034, r = −0.498). At AE-IPF, the A/L ratio was not related to PaO_2_/FiO_2_ (P/F) ratio (**e**; p = 0.979, r = −0.004) or HRCT extent score at AE (**f**; p = 0.269, r = −0.187). The A/L ratio was positively correlated with serum C-reactive protein (CRP) (**g**; p = 0.049, r = 0.316) and negatively correlated with serum KL-6 at AE (**h**; p = 0.035, r = −0.357). “PaO_2_ before AE” was evaluated within 12 months before AE-IPF.

The correlations between serum adiponectin/leptin levels at AE-IPF and several clinical parameters are shown in Fig. 2. There was no correlation between adiponectin and leptin levels (Fig. 2a). Next, relationships between serum adiponectin/leptin (A/L) ratio and clinical parameters were evaluated because adiponectin and leptin show vastly different serum concentrations in obesity and opposite effects on PPARγ expression^8^. A negative correlation was found between A/L ratio and BMI (Fig. 2b; p = 0.003, r = −0.456). The A/L ratio was negatively correlated with the period from IPF-diagnosis to AE (Fig. 2c; p = 0.042, r = −0.322) and PaO_2_ before AE (Fig. 2d; p = 0.034, r = −0.498). At AE-IPF, the A/L ratio showed no relationship with PaO_2_/FiO_2_ (P/F) ratio (Fig. 2e; p = 0.979, r = −0.004) or HRCT extent score at AE (Fig. 2f; p = 0.125, ρ = −0.260). Conversely, the A/L ratio was positively correlated with serum C-reactive protein (CRP) (Fig. 2g; p = 0.049, r = 0.316) and negatively correlated with serum KL-6 at AE (Fig. 2h; p = 0.035, r = −0.357). The A/L ratio showed no relationships with age (p = 0.545, r = −0.099), smoking pack years (p = 0.080, r = −0.280), %FVC (p = 0.711, r = −0.077), GAP stage (p = 0.301, ρ = −0.181), JRS severity stage (p = 0.937, ρ = −0.014), or serum surfactant protein-D (SP-D) level (p = 0.162, r = −0.242) (Supplementary Fig. S1).

### Comparisons of data between patients with higher and lower A/L ratio

Based on the median A/L ratio (median 905) at AE-IPF in 39 patients, the patients were divided into two groups. For the prognostic analysis, comparisons of clinical data between patients with higher A/L ratio (≥905) and lower A/L ratio (<905) in 32 patients without duplicate are shown in Supplementary Table S2. BMI was significantly lower in the higher A/L ratio group than in the lower group (p = 0.023). Regarding the severity of IPF before AE, FVC, JRS severity grades, or GAP stages were not significantly different between groups (p = 0.559, 0.205, and 0.626, respectively). The serum KL-6 level was significantly lower in the higher A/L ratio group than in the lower group (p = 0.022). The serum LDH and SP-D level also tended to be lower in the higher A/L ratio group than in the lower group. The proportion of patients with hyperlipidemia, diabetes mellitus, and administration of exogenous PPARγ agonists - which affect the biology of adiponectin and leptin - did not differ between groups. All patients received steroid pulse therapy, although fewer patients were administered immunosuppressants in the higher A/L ratio group than in the lower A/L ratio (p = 0.028). Other treatments and HRCT extent score did not differ between groups.

### Impact of serum A/L ratio on survival

**Figure 3:**
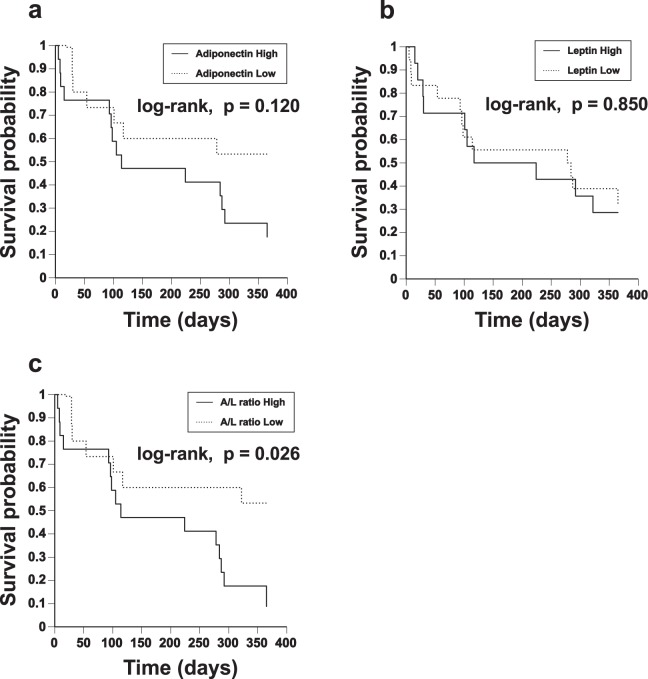
Impact of serum A/L ratio on survival. Among 32 patients with AE-IPF, Kaplan-Meier survival curves from the first AE-IPF onset are shown. When patients were divided into two groups based on median value of each adipokine, the 12-month survival rates were not significantly different between patients with higher and lower adiponectin or leptin (**a**; adiponectin, log-rank test, p = 0.120 and **b**; leptin, log-rank test, p = 0.850, respectively). The 12-month survival rate was significantly lower in patients with higher A/L ratio (≥905) than in those with lower A/L ratio (**c**, log-rank test, p = 0.026).

Among 32 patients with AE-IPF, seven died within 1 month of onset (mortality rate, 21.8%), 9 died within 3 months (mortality rate, 28.1%), and 23 died within 12 months (mortality rate, 71.8%) from the first episode of AE-IPF. Nineteen patients died of respiratory failure and four died of infectious pneumonia after beginning of steroid-treatment. Kaplan-Meier survival curves from the first AE-IPF onset are shown in Fig. 3. When patients were divided into two groups based on the median value of each adipokine, the 12-month survival rates were not significantly different among patients with higher and lower adiponectin or leptin (Fig. 3a; adiponectin, log-rank test, p = 0.120 and Fig. 3b; leptin, log-rank test, p = 0.850, respectively). However, the 12-month survival rate was significantly lower in patients with higher A/L ratios (≥905) than in those with lower A/L ratios (Fig. 3c, log-rank test, p = 0.026). The results of univariate Cox proportional hazards analyses for prognostic factors within 12 months of AE-IPF onset are shown in Supplementary Table S3. Serum adiponectin or leptin alone was not a significant prognostic factor (hazard ratio [HR] 1.000, p = 0.076 and HR 0.930, p = 0.075, respectively). However, A/L ratio itself and higher A/L ratio (≥905) were both significant factors of worse prognosis (HR 1.000, p = 0.006 and HR 2.729, p = 0.032, respectively). Additionally, peripheral blood neutrophils at AE (HR 1.000, p = 0.027) and period from admission to the beginning of AE treatment (HR 1.126, p = 0.032) were also significant prognostic factors. The results of multivariate Cox proportional hazards analyses, which show factors that predict survival adjusted for age, are shown in Table 2. A/L ratio itself and higher A/L ratio (≥905) were still significant factors of worse prognosis (HR 1.000, p = 0.012 and HR 2.600, p = 0.042, respectively), although adiponectin or leptin alone was not significant. Furthermore, JRS severity grade (HR 1.610, p = 0.040), preceding oxygen therapy (HR 2.628, p = 0.035), peripheral blood neutrophils at AE (HR 1.000, p = 0.020), and period from admission to the beginning of AE treatment (HR 1.125, p = 0.035) were also significant factors. Although A/L ratio was moderately and negatively associated with BMI (Fig. 2b), even in multivariate Cox proportional hazards analyses adjusted for BMI, A/L ratio itself and higher A/L ratio (≥905) were significant factors of worse prognosis (Supplementary Table S4; HR 1.000, p = 0.011 and HR 2.680, p = 0.046, respectively).

**Table 2:** Multivariate Cox Proportional Hazards models of survival adjusted for age.

Variable	Hazard Ratio	95% CI		p Value
		lower	upper	
Sex, male	0.231	0.047	1.125	ns.
Smoking pack years	0.985	0.967	1.004	ns.
Minimum SpO_2_ in 6MWT before AE*, %	0.932	0.866	1.003	ns.
JRS severity grade before AE*	1.610	1.023	1.535	0.040
Preceding oxygen therapy, +	2.628	1.072	6.440	0.035
Serum adiponectin, ng/mL	1.000	1.000	1.000	ns.
Serum leptin, ng/mL	0.927	0.855	1.005	ns.
Adiponectin/leptin ratio	1.000	1.000	1.000	0.012
Adiponectin/leptin ratio, ≥905	2.600	1.031	6.558	0.042
Peripheral blood neutrophils at AE, /μL	1.000	1.000	1.000	0.020
Serum KL-6 at AE, U/mL	1.000	0.999	1.000	ns.
P/F ratio at AE	0.996	0.992	1.001	ns.
Period from admission to the beginning of AE treatment, days	1.125	1.008	1.255	0.035

^*^Pulmonary function tests, severity scores, HRCT, and serum markers were evaluated within 12 months before AE-IPF.

Abbreviations; SpO_2_: partial oxygen saturation, 6MWT: 6-minute walk test, AE: acute exacerbation, JRS: Japanese respiratory society, KL-6: Krebs von den Lungen-6, P/F: PaO_2_/FiO_2,_ ns.: not significant.

## Discussion

In this study, we evaluated serum adipokine concentrations and determined whether adipokines were important to predict prognosis in patients with AE-IPF. Patients with AE-IPF had increased levels of serum adiponectin and leptin compared with those at first diagnosis of IPF. A/L ratio negatively correlated with BMI and PaO_2_ before AE-IPF, and positively correlated with CRP. Finally, A/L ratio was a significant prognostic factor in patients with AE-IPF. To the best of our knowledge, this is the first study to show the relationship between adiponectin and leptin, and the significance of these two adipokines in patients with AE-IPF.

Adiponectin and leptin are produced from adipose tissue, and these adipokines are related to obesity and type II diabetes mellitus^9^. In patients with obesity and type II diabetes mellitus, the serum adiponectin level decreases and that of leptin increases^9^. Conversely, in emaciated subjects, adiponectin increases and leptin decreases. Furthermore, the biological functions of both adipokines are closely linked to PPARγ activity and its anti-inflammation capacity^8,9^. In the current study, however, both adiponectin and leptin levels increased at AE-IPF, and the mechanism of action of these adipokines does not seem to be simple like in obesity or diabetes mellitus.

Leptin has proinflammatory effects via PPARγ suppression^8,9^. Leptin reportedly promotes profibrotic gene transcription related to transforming growth factor (TGF) *β* in patients with ARDS without obesity^12^. Further, Cao *et al*. reported that leptin levels increased at AE-IPF, and AE-IPF patients with higher leptin levels at AE had poorer prognosis than those with lower levels^13^. Even in the present study, the leptin level significantly increased at AE-IPF. However, higher leptin alone was not related to worse prognosis, and higher adiponectin rather seemed to be related to poor prognosis. Adiponectin has anti-inflammatory effects via activation of PPARγ^8,9^. Furthermore, adiponectin suppresses TGF *β* signaling centering on PPARγ^8^, and suppresses acute lung injury^14^. Reportedly, in patients with IPF^15^ or systemic sclerosis^10,11^, adiponectin levels significantly decreased. Conversely, several studies have shown that adiponectin has profibrotic effects. Higher adiponectin levels were positively related to advanced liver fibrosis in hepatitis B viral infection^16,17^, and lack of adiponectin was associated with dampened wound healing in diabetes mellitus^18^. Further, adiponectin promoted collagen production from fibroblast^19,20^, induced intercellular adhesion molecule (ICAM)-1 on fibroblast^21^, and then facilitated its adhesion capacity^20^. In the current study, both adipokines increased at AE-IPF, and higher A/L ratio was rather related to poorer prognosis. Although the exact mechanism related to these adipokines remains unknown in AE-IPF, these results imply that adiponectin’s anti-inflammatory (profibrotic) effects following leptin’s proinflammatory effects may play a role in the pathogenesis of AE-IPF. This pathogenesis seems to be similar to the activation of M2 (anti-inflammatory and profibrotic) alveolar macrophages following that of M1 (proinflammatory) macrophages in acute lung injury or ARDS^22^.

This study has several limitations. First, only a small number of patients with AE-IPF were enrolled. Second, the clinical and prognostic data were retrospectively analyzed. Finally, the treatment of AE-IPF was not uniform. A prospective and larger study should be conducted to determine the role of adipokines in patients with AE-IPF.

In conclusion, this study showed that serum adiponectin and leptin levels significantly increased in patients with AE-IPF. A/L ratio positively correlated with CRP, and A/L ratio was a significant prognostic factor in patients with AE-IPF. These adipokines are closely associated with the balance between inflammation and fibrosis, and may play a role in the pathogenesis of AE-IPF. A larger and prospective study is needed to precisely evaluate the role of these adipokines in the pathogenesis of AE-IPF.

## Methods

### Study design and patients

Thirty-two patients who were diagnosed with AE-IPF at our hospital between 1997 and 2016 were studied. Seven patients had two episodes of AE-IPF (Table 1). In sum, 39 episodes of AE, for which serum samples were available, were retrospectively reviewed. Twenty patients were thoroughly examined by surgical lung biopsy before developing AE-IPF and met the 2011 international consensus criteria for IPF^1^. The remaining 12 patients showed clinical and high-resolution computed tomography (HRCT) features identical to IPF and were diagnosed with IPF without surgical lung biopsy^1^. Patients diagnosed with any connective tissue disorders were excluded from the study.

AE-IPF was diagnosed according to the modified diagnostic criteria as previously described^23–26^. Patients enrolled in this study also met the 2016 AE-IPF criteria^27^. The study protocol was approved by the Ethical Committee of Hamamatsu University School of Medicine (approval number 18–085 and 15–165), and this study was carried out in accordance with the approved protocol. The need for patient approval and informed consent was waived due to the retrospective nature of the study.

### Data collection

Clinical data were obtained from the medical records. Disease severity of IPF within 12 months before the AE event was assessed using the GAP-staging system^28^ and the Japanese Respiratory Society (JRS)-severity scale of interstitial pneumonia^7^. The former considers gender, age, and two lung physiology variables: FVC and DL_CO_^28^. The latter is based on PaO_2_ at rest and minimum SpO_2_ during the 6-minute walking test (6MWT)^7^.

The extent of lung opacities was measured on three HRCT slices as previously described^29,30^. The sum of the scores from five lobes (0–25) was used to express the extent of lung opacities in each patient. The HRCT patterns of AE-IPF were evaluated as reported by Akira^31^. The HRCT findings were reviewed by two observers.

### Measurement of serum adiponectin and leptin concentrations

All blood samples were collected on the first or second day of admission before starting treatments for AE-IPF. Serum adiponectin and leptin concentrations were measured with enzyme-linked immunosorbent assay (R&D Systems, Inc., Minneapolis, MN, USA).

### Statistical analysis

Statistical analyses were performed using StatView J-4.5 and JMP-13.1.0 (SAS Institute Inc., Cary, NC, USA). Categorical data were compared using the Chi-square test or Fisher’s exact probability test for independence, and continuous data were compared using the unpaired *t*-test. Continuous data at different time points in the same patient were compared using the paired *t*-test. The relationship between adiponectin/leptin ratio and serial data was analyzed using Pearson’s correlation coefficient, and that between adiponectin/leptin ratio and discrete variable data was analyzed using Spearman’s rank correlation coefficient. Survival of patient groups was evaluated with Kaplan-Meier curves, and was compared between groups using the log-rank test. The relationships between variables and mortality were assessed by the Cox proportional hazards regression analysis. All tests were two-sided and statistical significance was set at p < 0.05.

## Supplementary information

Supplementary Information
