# Supplementary material for: Analysis of serum adiponectin and leptin in patients with acute exacerbation of idiopathic pulmonary fibrosis
Source: Sci Rep. 2019 Jul 19;9:10484. doi: 10.1038/s41598-019-46990-3 (PMC6642131; doi:10.1038/s41598-019-46990-3)
Supplement: Supplementary file 1 — Supplementary Information [file 41598_2019_46990_MOESM1_ESM.pdf]

Supplementary Information

TITLE: Analysis of serum adiponectin and leptin in patients with acute exacerbation of idiopathic pulmonary fibrosis

Authors' full names: Noriyuki Enomoto, M.D., Ph.D.[1, 2, *], Yoshiyuki Oyama, M.D.[1], Hideki Yasui, M.D., Ph.D.[1], Masato Karayama, M.D., Ph.D.[1], Hironao Hozumi, M.D., Ph.D.[1], Yuzo Suzuki, M.D., Ph.D.[1], Masato Kono, M.D., Ph.D.[1], Kazuki Furuhashi, M.D., Ph.D.[1], Tomoyuki Fujisawa, M.D., Ph.D.[1], Naoki Inui, M.D., Ph.D.[1, 3], Yutaro Nakamura, M.D., Ph.D.[1], Takafumi Suda, M.D., Ph.D.[1]

Authors' affiliation(s):
[1] Second Division, Department of Internal Medicine, Hamamatsu University School of Medicine, Hamamatsu, Japan
[2] Health Administration Center, Hamamatsu University School of Medicine, Hamamatsu, Japan
[3] Department of Clinical Pharmacology and Therapeutics, Hamamatsu University School of Medicine, Hamamatsu, Japan

Supplementary Figure S1. Relationships between serum adiponectin/leptin levels and clinical parameters other than data shown in Fig 2. The relationships between serum adiponectin/leptin (A/L) ratio at AE-IPF and other clinical parameters are shown. A/L ratio showed no relationships with age (a; p=0.545, r=-0.099), smoking pack years (b; p=0.080, r=-0.280), %FVC (c; p=0.711, r=-0.077), GAP stage (d; p=0.301, ρ =-0.181), JRS severity stage (e; p=0.937, ρ =-0.014) before AE, or serum SP-D level (f; p=0.162, r=-0.242). "%FVC, GAP stage, and JRS severity stage before AE" were evaluated within 12 months before AE-IPF.

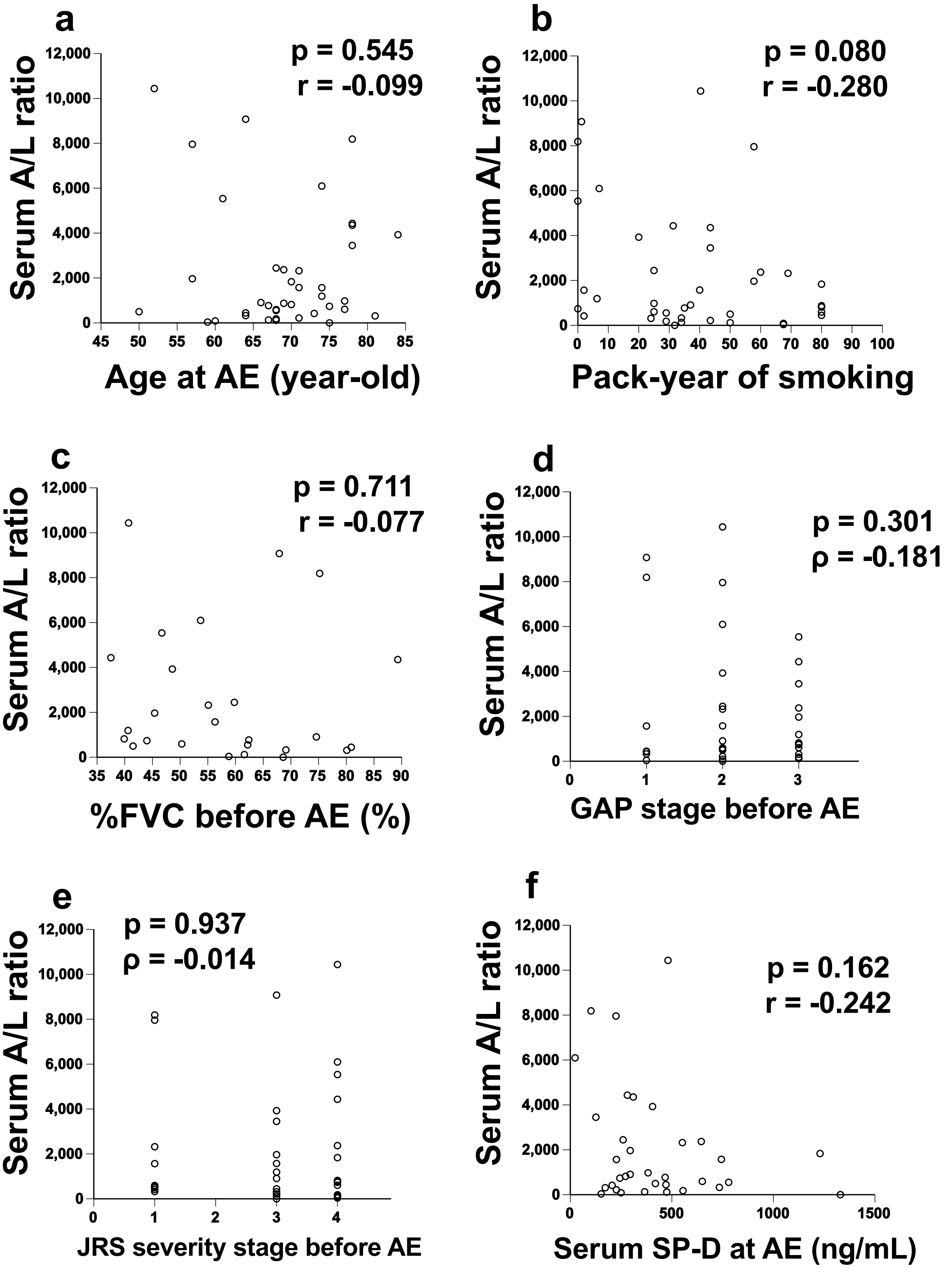

Supplementary Table S1. Clinical characteristics, severity, and treatments for IPF and/or AE-IPF (others)

	**n=39 episodes, median (range)**
Diagnosis of IPF, surgical lung biopsy / clinical, n	20 / 19
Hyperlipidemia, + / -	5 / 35
Diabetes mellitus, + / -	16 / 24
Exogenous PPARγ agonist (e.g. pioglitazone, ARB), + / -	9 / 31
FEV1 / FVC before AE, %	87.9 (78.5, 107)
Serum LDH within 3 months before AE, IU/L	273 (168, 451)
Serum KL-6 within 3 months before AE, U/mL	1170 (400, 3570)
Serum SP-D within 3 months before AE, ng/mL	255 (86, 531)
HRCT pattern of AE, peripheral / multifocal / diffuse	3 / 2 / 32
Treatment with PMX-DHP, + / -	20 / 20

Abbreviations; IPF: idiopathic pulmonary fibrosis, AE: acute exacerbation, PPARγ: peroxisome proliferator-activated receptor, ARB: angiotensin Ⅱ receptor blocker, FEV1: forced expiratory volume in one second, FVC: forced vital capacity, LDH: lactate dehydrogenase, KL-6: Krebs von den Lungen-6, SP-D: surfactant protein D, HRCT: high-resolution computed tomography, PMX-DHP: direct haemoperfusion with a polymyxin B-immobilised fibre column.

Supplementary Table S2. Comparison of data between patients with high and low adiponectin/leptin ratios

	**Adiponectin/Leptin ratio <905** (n=15,median (range))	**Adiponectin/Leptin ratio ≥905** (n=17,median (range))	**p value**
Age, years	68 (50, 81)	71 (52, 84)	ns.
Sex, male / female	14 / 1	16 / 1	ns.
Smoking, never / ex / current	1 / 12 / 2	2 / 13 / 2	ns.
Smoking pack years	43.5 (0, 80)	31.3 (0, 80)	ns.
Period from IPF-diagnosis to AE, mo	100 (1, 203)	55 (0, 146)	ns.
The number of AEs, 1 / 2	14 / 1	16 / 1	ns.
Observation period from the onset of AE, days	95 (2, 205)	35 (4, 139)	0.029
BMI	23.0 (20.2, 28.9)	20.7 (16.3, 27.7)	0.023
Body surface area, m^2	1.7 (1.4, 1.9)	1.5 (1.4, 1.9)	ns.
Hyperlipidemia, + / -	3 / 12	1 / 16	ns.
Diabetes mellitus, + / -	7 / 8	5 / 12	ns.
Exogenous PPARγ agonist (e.g. pioglitazone, ARB), + / -	5 / 10	1 / 16	ns.
Peripheral blood WBC at AE, $\times 10^3$/μL	8.3 (1.9, 17.6)	10.3 (5.5, 20.0)	ns.
Peripheral blood neutrophils at AE, $\times 10^3$/μL	6.2 (1.5, 15.3)	7.5 (5.0, 18.2)	ns.
Serum CRP at AE, mg/dL	5.5 (1.1, 23.7)	11.3 (0.9, 21.9)	ns.
Serum LDH at AE, IU/L	365 (220, 601)	319 (183, 464)	ns.
Serum KL-6 at AE, U/mL	2320 (1034, 6404)	1122 (481, 2640)	0.022
Serum SP-D at AE, ng/mL	443 (152, 1330)	347 (23, 1230)	ns.
P/F ratio at AE	192 (38, 386)	167 (44, 368)	ns.
HRCT extent scores at AE (full score: 25)	20.5 (14, 25)	19 (13, 22)	ns.
HRCT pattern at AE, peripheral / multifocal / diffuse / unknown	1 / 1 / 12 / 1	2 / 1 / 12 / 2	ns.
Period from admission to the beginning of AE treatment, days	1 (0, 17)	0 (0, 6)	ns.
Administration of steroid pulse therapy, + / -	15 / 0	17 / 0	ns.
Administration of Sivelestat sodium hydrate, + / -	3 / 12	4 / 13	ns.
Administration of Immunosuppressant, + / -	13 / 2	8 / 9	0.028
Treatment with PMX-DHP, + / -	9 / 6	7 / 10	ns.
Intubation at AE, + / -	3 / 12	5 / 12	ns.
ΔLDH at 2 days after beginning AE treatment, IU/L	-54 (-94, 307)	-32.5 (-145, 425)	ns.
ΔP/F ratio at 2 days after beginning AE treatment	40.0 (-146, 200)	-10.0 (-92.0, 128)	ns.
**Data before AE ***			
PaO_2 at rest, Torr	78.0 (52.0, 91.0)	71.4 (49.0, 79.0)	ns.
Distance in 6MWT, m	365 (160, 507)	375 (170, 465)	ns.
Minimum SpO_2 in 6MWT, %	82 (60, 95)	83 (73, 87)	ns.
HRCT extent scores (full score: 25)	12.5 (7, 19)	12 (8, 16)	ns.
FVC, % pred	61.9 (39.9, 80.9)	54.4 (37.5, 89.3)	ns.
FEV1 / FVC before AE *, %	87.3 (78.5, 107.0)	87.9 (79.7, 98.7)	ns.
DL_{CO} before AE *, % pred	73.9 (47.4, 79.8)	50.0 (33.5, 85.9)	ns.
JRS severity grade, Ⅰ / Ⅱ / Ⅲ / Ⅳ / unknown	5 / 0 / 5 / 4 / 1	2 / 0 / 6 / 6 / 3	ns.
The GAP staging system, Ⅰ / Ⅱ / Ⅲ / unknown	4 / 5 / 5 / 1	2 / 6 / 5 / 4	ns.
Preceding treatments for IPF, + / -	7 / 8	10 / 7	ns.
Preceding oxygen therapy, + / -	6 / 9	4 / 13	ns.

* Pulmonary function tests, severity scores, HRCT, and serum markers were evaluated within 12 months before AE-IPF.

Abbreviations; IPF: idiopathic pulmonary fibrosis, AE: acute exacerbation, BMI: body mass index, PPAR: peroxisome proliferator-activated receptor, PaO_2: partial pressure of arterial oxygen, 6MWT: 6-minute walk test, SpO_2: partial oxygen saturation, HRCT: high-resolution computed tomography, PMX-DHP: direct haemoperfusion with a polymyxin B-immobilised fibre column, FVC: forced vital capacity, FEV1: forced expiratory volume in one second, DL_{CO}: diffusion lung capacity for carbon monoxide, JRS: Japanese respiratory society, GAP: gender, age, and physiology, WBC: white blood cell, CRP: c-reactive protein, LDH: lactate dehydrogenase, KL-6: Krebs von den Lungen-6, SP-D: surfactant protein D, P/F: PaO_2/FiO, ns.: not significant.

Supplementary Table S3. Univariate Cox Proportional Hazards models of survival

Variable	Hazard ratio	95% CI		p value
		lower	upper	
Age, yr	1.030	0.964	1.100	ns.
Sex, male	0.272	0.058	1.270	ns.
Smoking pack years	0.984	0.967	1.002	ns.
Period from IPF-diagnosis to AE, mo	0.998	0.991	1.006	ns.
BMI	0.941	0.814	1.089	ns.
Body surface area, m^2	0.194	0.011	3.300	ns.
Exogenous PPARγ agonist (e.g. pioglitazone, ARB), +	0.515	0.152	1.742	ns.
FVC before AE *, % pred	0.983	0.946	1.021	ns.
FEV_1 / FVC before AE *, %	0.970	0.896	1.049	ns.
DL_{CO} before AE *, % pred	0.984	0.950	1.019	ns.
Resting PaO_2 before AE *, mmHg	0.993	0.949	1.039	ns.
Distance in 6MWT before AE *, m	0.995	0.989	1.002	ns.
Minimum SpO_2 in 6MWT before AE *, %	0.937	0.872	1.008	ns.
JRS severity grade before AE *	1.528	0.994	2.347	ns.
The GAP staging system before AE *	1.340	0.712	2.522	ns.
Extent score on HRCT before AE *	1.096	0.945	1.272	ns.
Preceding treatments for IPF, +	0.903	0.398	2.048	ns.
Preceding oxygen therapy, +	2.318	0.970	5.537	ns.
Serum adiponectin, ng/mL	1.000	1.000	1.000	ns.
Serum leptin, ng/mL	0.930	0.858	1.008	ns.
Adiponectin/leptin ratio	1.000	1.000	1.000	0.006
Adiponectin/leptin ratio, ≥905	2.729	1.093	6.815	0.032
Peripheral blood WBC at AE, /µL	1.000	1.000	1.000	ns.
Peripheral blood neutrophils at AE, /µL	1.000	1.000	1.000	0.027
Serum CRP at AE, mg/dL	1.034	0.975	1.097	ns.
Serum LDH at AE, IU/L	1.000	0.994	1.006	ns.
Serum KL-6 at AE, U/mL	1.000	0.999	1.000	ns.
Serum SP-D at AE, ng/mL	0.999	0.997	1.001	ns.
P/F ratio at AE	0.996	0.992	1.001	ns.
Extent score on HRCT at AE	1.065	0.950	1.195	ns.
Period from admission to the beginning of AE treatment, days	1.126	1.010	1.255	0.032

* Pulmonary function tests, severity scores, HRCT, and serum markers were evaluated within 12 months before AE-IPF.

Abbreviations; IPF: idiopathic pulmonary fibrosis, AE: acute exacerbation, BMI: body mass index, PPAR: peroxisome proliferator-activated receptor, FVC: forced vital capacity, FEV1: forced expiratory volume in one second, DL_{CO}: diffusion lung capacity for carbon monoxide, PaO_2: partial pressure of arterial oxygen, 6MWT: 6-minute walk test, SpO_2: partial oxygen saturation, JRS: Japanese respiratory society, GAP: gender, age, and physiology, HRCT: high-resolution computed tomography, WBC: white blood cell, CRP: c-reactive protein, LDH: lactate dehydrogenase, KL-6: Krebs von den Lungen-6, SP-D: surfactant protein D, P/F: PaO_2/FiO_2, ns.: not significant..

Supplementary Table S4. Multivariate Cox Proportional Hazards models of survival adjusted for BMI

Variable	Hazard Ratio	95% CI		p Value
		lower	upper	
Serum adiponectin, ng/mL	1.000	1.000	1.000	ns.
Serum leptin, ng/mL	0.934	0.860	1.015	ns.
Adiponectin/leptin ratio	1.000	1.000	1.000	0.011
Adiponectin/leptin ratio, ≥905	2.680	1.017	7.060	0.046

Abbreviations; BMI: body mass index, ns.: not significant.
